# Supplementary material for: Surface texture limits transfer of S. aureus, T4 bacteriophage, influenza B virus and human coronavirus
Source: PLoS One. 2020 Dec 28;15(12):e0244518. doi: 10.1371/journal.pone.0244518 (PMC7769612; doi:10.1371/journal.pone.0244518)
Supplement: S1 Table — (DOCX) [file pone.0244518.s001.docx]

**S1 Table. Measurements of *S. aureus* transfer on silicone surfaces using the bead transfer method.**

|  |  | **Log10-transformed CFU/mL** | | | **CFU/mL** | | | |  |  | | |
| --- | --- | --- | --- | --- | --- | --- | --- | --- | --- | --- | --- | --- |
| **Assay #** | **Operator** | **Smooth** | | **+3SK2x2** | | **Smooth** | | **+3SK2x2** | **Inoculum (PFU/mL)** | | | **Log inoculum** |
| 1 | 1 | 1.96 | | 0.30 | | 8.37E+02 | | 5.90E-06 | 4.90E+03 | | | 3.69 |
| 2 | 1 | 2.42 | | 0.00 | | 6.89E+03 | | 0.00E+00 | 5.65E+03 | | | 3.75 |
| 3 | 1 | 2.17 | | 0.00 | | 2.32E+03 | | 0.00E+00 | 5.65E+03 | | | 3.75 |
| 4 | 2 | 2.25 | | 1.04 | | 3.33E+03 | | 1.48E+00 | 2.72E+03 | | | 3.43 |
| 5 | 2 | 2.73 | | 1.08 | | 2.30E+04 | | 2.16E+00 | 3.65E+03 | | | 3.56 |
| 6 | 2 | 2.60 | | 1.18 | | 1.41E+04 | | 5.23E+00 | 3.65E+03 | | | 3.56 |
| 7 | 3 | 2.36 | | 0.78 | | 5.36E+03 | | 8.34E-02 | 3.10E+03 | | | 3.49 |
| 8 | 3 | 2.39 | | 1.00 | | 6.08E+03 | | 1.00E+00 | 3.21E+03 | | | 3.51 |
| 9 | 3 | 2.56 | | 0.78 | | 1.21E+04 | | 8.34E-02 | 3.21E+03 | | | 3.51 |
|  |  |  | |  | |  | |  |  | | |  |
| **Average** | | **2.38** | **0.68** | | | **8.22E+03** | **1.12E+00** | | **3.97E+03** | | **3.58** | |
| **Log Reduction** | |  | **1.70** | | |  |  | |  | |  | |
| **% Reduction** | |  | **98.0%** | | |  |  | |  | |  | |
