# Supplementary material for: Surface texture limits transfer of S. aureus, T4 bacteriophage, influenza B virus and human coronavirus
Source: PLoS One. 2020 Dec 28;15(12):e0244518. doi: 10.1371/journal.pone.0244518 (PMC7769612; doi:10.1371/journal.pone.0244518)
Supplement: S2 Table — (DOCX) [file pone.0244518.s002.docx]

**S2 Table. Measurements of T4 bacteriophage transfer on silicone surfaces using the bead transfer method.**

|  |  | **Log10-transformed PFU/mL** | | **PFU/mL** | |  | |  |
| --- | --- | --- | --- | --- | --- | --- | --- | --- |
| **Assay #** | **Operator** | **Smooth** | **+3SK2x2** | **Smooth** | **+3SK2x2** | **Inoculum (PFU/mL)** | **Log inoculum** | |
| 1 | 3 | 3.46 | 2.03 | 2.49E+05 | 1.18E+03 | 4.55E+06 | 6.66 | |
| 2 | 2 | 3.64 | 1.57 | 4.06E+05 | 9.00E+01 | 1.62E+06 | 6.21 | |
| 3 | 1 | 3.73 | 2.79 | 5.20E+05 | 2.90E+04 | 3.07E+06 | 6.49 | |
| 4 | 1 | 3.73 | 2.92 | 5.18E+05 | 4.45E+04 | 2.62E+06 | 6.42 | |
| 5 | 3 | 3.73 | 2.86 | 5.19E+05 | 3.60E+04 | 3.55E+06 | 6.55 | |
| 6 | 2 | 3.20 | 1.75 | 1.11E+05 | 2.65E+02 | 1.34E+06 | 6.13 | |
| 7 | 2 | 3.76 | 2.60 | 5.58E+05 | 1.39E+04 | 1.65E+06 | 6.22 | |
| 8 | 1 | 3.25 | 2.36 | 1.31E+05 | 5.41E+03 | 2.55E+06 | 6.41 | |
| 9 | 3 | 3.88 | 3.11 | 7.67E+05 | 8.39E+04 | 4.55E+06 | 6.66 | |
|  |  |  |  |  |  |  |  | |
| **Average** | | **3.60** | **2.44** | **4.20E+05** | **2.38E+04** | **2.83E+06** | **6.41** | |
| **Log Reduction** | |  | **1.15** |  |  |  |  | |
| **% Reduction** | |  | **93.0%** |  |  |  |  | |
