# Supplementary material for: Surface texture limits transfer of S. aureus, T4 bacteriophage, influenza B virus and human coronavirus
Source: PLoS One. 2020 Dec 28;15(12):e0244518. doi: 10.1371/journal.pone.0244518 (PMC7769612; doi:10.1371/journal.pone.0244518)
Supplement: S3 Table — (DOCX) [file pone.0244518.s003.docx]

**S3 Table. Measurements of human virus transfer on silicone surfaces using the bead transfer method.**

| **Influenza B Virus** | | **Log10-transformed TCID50** | | | **TCID50** | | |
| --- | --- | --- | --- | --- | --- | --- | --- |
| **Assay #** | **Operator** | Smooth | SK2x2 | SK5x3 | Smooth | SK2x2 | SK5x3 |
| 1 | 1 | 4.22 | 3.88 | 3.66 | 1.79E+06 | 7.73E+05 | 4.31E+05 |
| 2 | 1 | 4.23 | 3.79 | 3.77 | 1.83E+06 | 6.11E+05 | 5.80E+05 |
| 3 | 1 | 4.55 | 4.01 | 4.12 | 3.80E+06 | 1.08E+06 | 1.41E+06 |
| 4 | 1 | 4.39 | 3.75 | 3.77 | 2.66E+06 | 5.50E+05 | 5.80E+05 |
| 5 | 2 | 4.24 | 3.84 | 4.05 | 1.88E+06 | 6.97E+05 | 1.19E+06 |
| 6 | 2 | 4.59 | 3.94 | 3.49 | 4.15E+06 | 9.01E+05 | 2.68E+05 |
| 7 | 2 | 4.31 | 4.10 | 4.06 | 2.21E+06 | 1.34E+06 | 1.22E+06 |
|  |  |  |  |  |  |  |  |
| **Average** | | **4.36** | **3.90** | **3.85** | **2.62E+06** | **8.50E+05** | **8.10E+05** |
| **Log Reduction** | |  | **0.46** | **0.52** |  |  |  |
| **% Reduction** | |  | **65.3%** | **69.5%** |  |  |  |
|  |  |  |  |  |  |  |  |
| **Coronavirus 229E** | | **Log10-transformed TCID50** | | | **TCID50** | | |
| **Assay #** | **Operator** | Smooth | SK2x2 | SK5x3 | Smooth | SK2x2 | SK5x3 |
| 1 | 1 | 2.90 | 2.61 | 2.48 | 4.21E+04 | 1.47E+04 | 8.80E+03 |
| 2 | 1 | 2.86 | 2.44 | 2.19 | 3.66E+04 | 7.48E+03 | 2.54E+03 |
| 3 | 1 | 2.75 | 2.14 | 2.57 | 2.47E+04 | 2.01E+03 | 1.26E+04 |
| 4 | 1 | 2.90 | 2.27 | 2.38 | 4.21E+04 | 3.63E+03 | 5.83E+03 |
| 5 | 2 | 2.63 | 2.09 | 2.49 | 1.58E+04 | 1.59E+03 | 9.16E+03 |
| 6 | 2 | 2.75 | 2.52 | 2.50 | 2.47E+04 | 1.03E+04 | 9.54E+03 |
| 7 | 2 | 2.82 | 2.24 | *1.77^a^* | 3.18E+04 | 3.18E+03 | *3.02E+02^a^* |
| 8 | 2 | 2.77 | 2.49 | 2.48 | 2.66E+04 | 9.16E+03 | 8.80E+03 |
|  |  |  |  |  |  |  |  |
| **Average** | | **2.80** | **2.35** | **2.44** | **3.06E+04** | **6.51E+03** | **8.18E+03** |
| **Log Reduction** | |  | **0.45** | **0.36** |  |  |  |
| **% Reduction** | |  | **64.3%** | **56.0%** |  |  |  |

^a^Numbers in red italics were identified as outliers by the ROUT method using Prism 8 and excluded from analysis.
