# Supplementary material for: Surface texture limits transfer of S. aureus, T4 bacteriophage, influenza B virus and human coronavirus
Source: PLoS One. 2020 Dec 28;15(12):e0244518. doi: 10.1371/journal.pone.0244518 (PMC7769612; doi:10.1371/journal.pone.0244518)
Supplement: S4 Table — (DOCX) [file pone.0244518.s004.docx]

**S4 Table. Measurements of human virus transfer on polypropylene surfaces using the bead transfer method.**

| **Influenza B virus** |  | **Log10-transformed TCID50** | | **TCID50** | |
| --- | --- | --- | --- | --- | --- |
| **Assay #** | **Operator** | Smooth | SK2x2 | Smooth | SK2x2 |
| 1 | 1 | 3.43 | 2.80 | 2.26E+05 | 2.91E+04 |
| 2 | 1 | 3.54 | 3.01 | 3.10E+05 | 6.07E+04 |
| 3 | 1 | 3.96 | 3.18 | 9.59E+05 | 1.06E+05 |
| 4 | 2 | 4.09 | 2.83 | 1.32E+06 | 3.35E+04 |
| 5 | 2 | 3.96 | 3.16 | 9.54E+05 | 9.89E+04 |
| 6 | 2 | 3.63 | 3.06 | 3.94E+05 | 7.23E+04 |
|  |  |  |  |  |  |
| **Average** | | **3.77** | **3.01** | **6.94E+05** | **6.68E+04** |
| **Log Reduction** | |  | **0.76** |  |  |
| **% Reduction** | |  | **82.8%** |  |  |
|  |  |  |  |  |  |
| **Coronavirus 229E** |  | **Log10-transformed TCID50** | | **TCID50** | |
| **Assay #** | **Operator** | Smooth | SK2x2 | Smooth | SK2x2 |
| 1 | 1 | 2.91 | 2.02 | 4.33E+04 | 1.15E+03 |
| 2 | 1 | 2.54 | 1.90 | 1.12E+04 | 6.09E+02 |
| 3 | 1 | 3.04 | 2.48 | 6.82E+04 | 8.77E+03 |
| 4 | 2 | 3.16 | 2.32 | 9.93E+04 | 4.52E+03 |
| 5 | 2 | 3.04 | 2.08 | 6.74E+04 | 1.52E+03 |
| 6 | 2 | 3.02 | 1.95 | 6.31E+04 | 7.95E+02 |
|  |  |  |  |  |  |
| **Average** | | **2.95** | **2.13** | **5.88E+04** | **2.89E+03** |
| **Log Reduction** | |  | **0.83** |  |  |
| **% Reduction** | |  | **85.1%** |  |  |
