# Supplementary material for: Surface texture limits transfer of S. aureus, T4 bacteriophage, influenza B virus and human coronavirus
Source: PLoS One. 2020 Dec 28;15(12):e0244518. doi: 10.1371/journal.pone.0244518 (PMC7769612; doi:10.1371/journal.pone.0244518)
Supplement: Attachment — Submitted filename: Response to Reviewers.pdf [file pone.0244518.s005.pdf]

Sharklet®
Technologies, Inc.

11/25/2020

Response to review of PONE-D-20-30941

Dear Dr. Mukherjee:

I would like to thank you and the reviewers for your consideration and comments on the manuscript entitled "Surface texture limits transfer of *S. aureus,* T4 Bacteriophage, Influenza B virus and Human coronavirus." We have found the reviews very helpful and resubmit with the following changes to the draft:

Editor comments:

1. Please ensure that your manuscript meets PLOS ONE's style requirements, including those for file naming.
    a. The manuscript has been updated to reflect the appropriate style.

2. Thank you for stating the following in the Financial Disclosure section:

a. Please provide an amended Funding Statement declaring this commercial affiliation, as well as a statement regarding the Role of Funders in your study. If the funding organization did not play a role in the study design, data collection and analysis, decision to publish, or preparation of the manuscript and only provided financial support in the form of authors' salaries and/or research materials, please review your statements relating to the author contributions, and ensure you have specifically and accurately indicated the role(s) that these authors had in your study. You can update author roles in the Author Contributions section of the online submission form.

Please also include the following statement within your amended Funding Statement.

"The funder provided support in the form of salaries for authors [insert relevant initials], but did not have any additional role in the study design, data collection and analysis, decision to publish, or preparation of the manuscript. The specific roles of these authors are articulated in the 'author contributions' section."

If your commercial affiliation did play a role in your study, please state and explain this role within your updated Funding Statement.

b. Please also provide an updated Competing Interests Statement declaring this commercial affiliation along with any other relevant declarations relating to employment, consultancy, patents, products in development, or marketed products, etc.

Within your Competing Interests Statement, please confirm that this commercial affiliation does not alter your adherence to all PLOS ONE policies on sharing data and materials by including

the following statement: "This does not alter our adherence to PLOS ONE policies on sharing data and materials." (as detailed online in our guide for authors http://journals.plos.org/plosone/s/competing-interests). If this adherence statement is not accurate and there are restrictions on sharing of data and/or materials, please state these. Please note that we cannot proceed with consideration of your article until this information has been declared.

c. Please include both an updated Funding Statement and Competing Interests Statement in your cover letter. We will change the online submission form on your behalf.

We apologize for any confusion regarding the funding and competing interests associated with this submission. We attempted to clearly communicate that all authors were employees of Sharklet Technologies, Inc while working on the manuscript. Please accept the following statements to reflect the requested changes:

Amended Funding Statement

This work was funded by internal Sharklet Technologies, Inc. funding. All authors were employees of Sharklet Technologies, Inc. while contributing to this study. The funder provided support in the form of salaries for authors [QL, LB, AH, JC, AB, and CJ] and test materials, but did not have any additional role in the study design, data collection and analysis, decision to publish, or preparation of the manuscript. The specific roles of these authors are articulated in the 'author contributions' section.

Amended Competing Interests Statement

I have read the journal's policy and the authors of this manuscript have the following competing interest: all authors were employed by Sharklet Technologies while contributing to this manuscript. Additionally, Anthony B. Brennan is a member of the Sharklet Technologies, Inc. Board of Directors. This does not alter our adherence to PLOS ONE policies on sharing data and materials.

Reviewer #1: This manuscript described a method using bead transfer to prevent fomite transmission. The presented method is useful and adaptable to many public settings. Yet the figures are very poor, but they only provided plots which is not enough. More evidence is needed to support their conclusions, for example, CFU numbers etc. Therefore, the manuscript may be acceptable if the authors can provide more results (not just graphs) to testify the transfer in S. aureus, T4 Bacteriophage, Influenza B virus and Human coronavirus.

Response to Reviewer #1: Thank you for your comments. We have added four Supplementary Tables to the submission including the raw data for each of the microbial experiments.

Reviewer #2: The manuscript deals with application of the Sharklet surface which is already a proven technology wherein inhibition of adhesion of microbes is known. However it is interesting

to observe that percentage reduction of human coronavirus on these surfaces ranges from 63 - 67%. The T4 bacteriophages have performed better. The data in this manuscript is of high importance and the manuscript well written. I commend the authors and strongly recommend for publication.

Response to Reviewer #2: Thank you for the positive comments regarding the manuscript. We agree that this is an important finding in the struggle against surface contamination across a wide range of infectious agents.

This manuscript has been posted to MedRxiv and has been assigned the DOI: 10.1101/2020.09.10.20192351.

We thank you for considering the resubmission of this manuscript. We believe that we have addressed each of the comments sufficiently and look forward to your response.

If I can be of any more assistance, please do not hesitate to contact me.

Sincerely,

Christopher J. Jones, PhD
Director of Research and Development
Sharklet Technologies, Inc.
cjones@sharklet.com
